# A database of elemental compositions of architectural float glass samples measured by LA-ICP-MS

**DOI:** 10.1016/j.dib.2020.105449

**Published:** 2020-03-18

**Authors:** Soyoung Park, Alicia Carriquiry, L. Kenneth Horkley, David W. Peate

**Affiliations:** a Department of Statistics, Iowa State University, 195 Durham, Ames, IA 50011, United States; b Materials, Analysis, Testing and Fabrication Facility, University of Iowa, 198 Iowa Advanced Technology Building, 205 N Madison St., Iowa City, IA 52242, United States; c Department of Earth & Environmental Sciences, University of Iowa, 115 Trowbridge Hall, Iowa City, IA 52242, United States

**Keywords:** Trace evidence, Float glass analysis, Chemical compositions, LA-ICP-MS, Forensic analysis, Statistics

## Abstract

We measured the elemental chemical composition of architectural float glass fragments using inductively coupled mass spectrometry with a laser ablation add-in. Measurements of 18 elemental concentrations were obtained from each fragment at each measurement occasion. These data can be used for statistical analysis with the purpose of evaluating forensic trace evidence. The data collection and measurement process in this database were carefully designed by the authors to enable understanding similarities and differences in elemental composition within a fragment, between fragments within a pane, between panes produced by the same manufacturer, and between manufacturers, to help in forensic glass evaluation. We received 48 panes that were produced on consecutive days, from two glass manufacturers in the U.S. Half of each pane was broken into small fragments and 24 fragments were randomly sampled from each half pane. To compute well-conditioned estimates of high-dimensional covariance matrices at all levels, we replicated measurements on each fragment; for three of the 24 fragments from a pane, we obtained 20 replicate measurements, and for the other 21 fragments, we made five replicate measurements. Analytical procedures to carry out the measurements followed the protocols recommended for forensic float glass samples by ENFSI [1] and the ASTM [2]. The database described in this article is related to two published research articles, “Learning algorithms to evaluate forensic glass evidence” by Park and Carriquiry (2019) [3] and “Evaluation and comparison of methods for forensic glass source conclusions” by Park and Tyner (2019) [4].

Specifications table

**Table:** 

Subject	Analytical Chemistry
Specific subject area	*Spectroscopy, Statistics, Probability and Uncertainty*
Type of data	Excel files
How data were acquired	Elemental chemical composition in glass fragments was measured by LA-ICP-MS. The unit for all measurements is parts per million (µg g^−1^). Instruments: Laser-ablation inductively-coupled plasma mass spectrometry (LA-ICP-MS) using a 213 nm NewWave laser ablation unit coupled to a Thermo X-series II ICP-MS. Data were processed using Iolite software
Data format	Raw
Parameters for data collection	Statistical analysis was conducted to understand the variability in the chemical composition of float glass at several levels: between fragments, between panes, between manufacturers. Understanding variability in chemical composition is necessary to answer questions about the source of a fragment found at a crime scene or on a suspect. Each measurement consists of an 18-dimensional vector. The elements we considered were: *Ca, Na, Mg, Al, K, Fe, Li, Ti, Mn, Rb, Sr, Zr, Ba, La, Ce, Nd, Hf,* and *Pb.* Each pane was broken into two parts. Half of the pane was broken to produce many random-sized fragments. Among broken fragments, 24 fragments were randomly sampled for chemical analysis. Five replicated measurements were obtained from 21 fragments and 20 replicated measurements were obtained from the rest of the fragments from each half pane.
Description of data collection	Float glass samples (panes) were donated by two glass manufacturers in the United States. The database includes 31 panes from company A (labeled AA, AB, …, AAR) and 17 panes from company B (labeled BA, BB, …, BR). Glass samples were obtained every day between Monday and Friday over a three-week period by manufacturer A, and over a two-week period by manufacturer B. On each day, a 5″x5″ pane of glass was obtained from the right side of the ribbon and another pane was cut from the left side of the ribbon.
Data source location	City/Town/Region: Ames/ Iowa and Iowa City/ Iowa Country: United States
Data accessibility	Repository name: Elemental chemical compositions of float glass samples Direct URL to data: https://doi.org/10.25380/iastate.11794368.v1
Related research article	Park, S. and Carriquiry, A., 2019. Learning algorithms to evaluate forensic glass evidence. *The Annals of Applied Statistics*, 13(2), pp.1068–1102, doi:10.1214/18-AOAS1211. Park, S. and Tyner, S., 2019. Evaluation and comparison of methods for forensic glass source conclusions. *Forensic science international*, 305, 110003, doi:10.1016/j.forsciint.2019.110003.

## Value of the data

• The sampling design is unique in that it permits estimation of the joint variability of elemental concentrations within fragments, between fragments within the pane, between pane within the manufacturer, and between manufacturers from nearly 8000 measurements from approximately 1200 fragments.
• The float glass panes analysed were manufactured on consecutive days, so the data allow investigation of changes in the chemical composition of the glass over time for each manufacturer. The data also provide a glimpse into the value of float glass evidence for use in criminal investigations: what is the production window within which two glass panes cannot be distinguished?
• Three out of 24 fragments were repeatedly measured 20 times. Therefore, these data can be used to obtain positive-definite sample covariance estimates in 18 dimensions for about 90 fragments manufactured by company A and about 60 fragments manufactured by B.
• The database of chemical concentrations of float glass panes is useful for forensic scientists working on trace evidence, for chemists interested in elemental analyses and for metrologists with interest in measurement error and reliability of ICP-MS. The large database could be used for training forensic examiners and for educating students studying forensic science.
• The data collection and measurement process in this database were carefully designed by the authors to include a wealth of replications at all levels that permit computing well-conditioned estimates of high-dimensional covariance matrices. The dataset is unique in that regard and we hope that it will be a good example for future data collection projects by other forensic scientists.
• Because of the design of the sample, the dataset will be a valuable resource for anyone working on methods for estimating and comparing covariance matrices, anyone who is interested in shrinkage methods and anyone who wishes to develop methods for the analyses of multivariate data.

## 1 Data

**Fig. 1:**
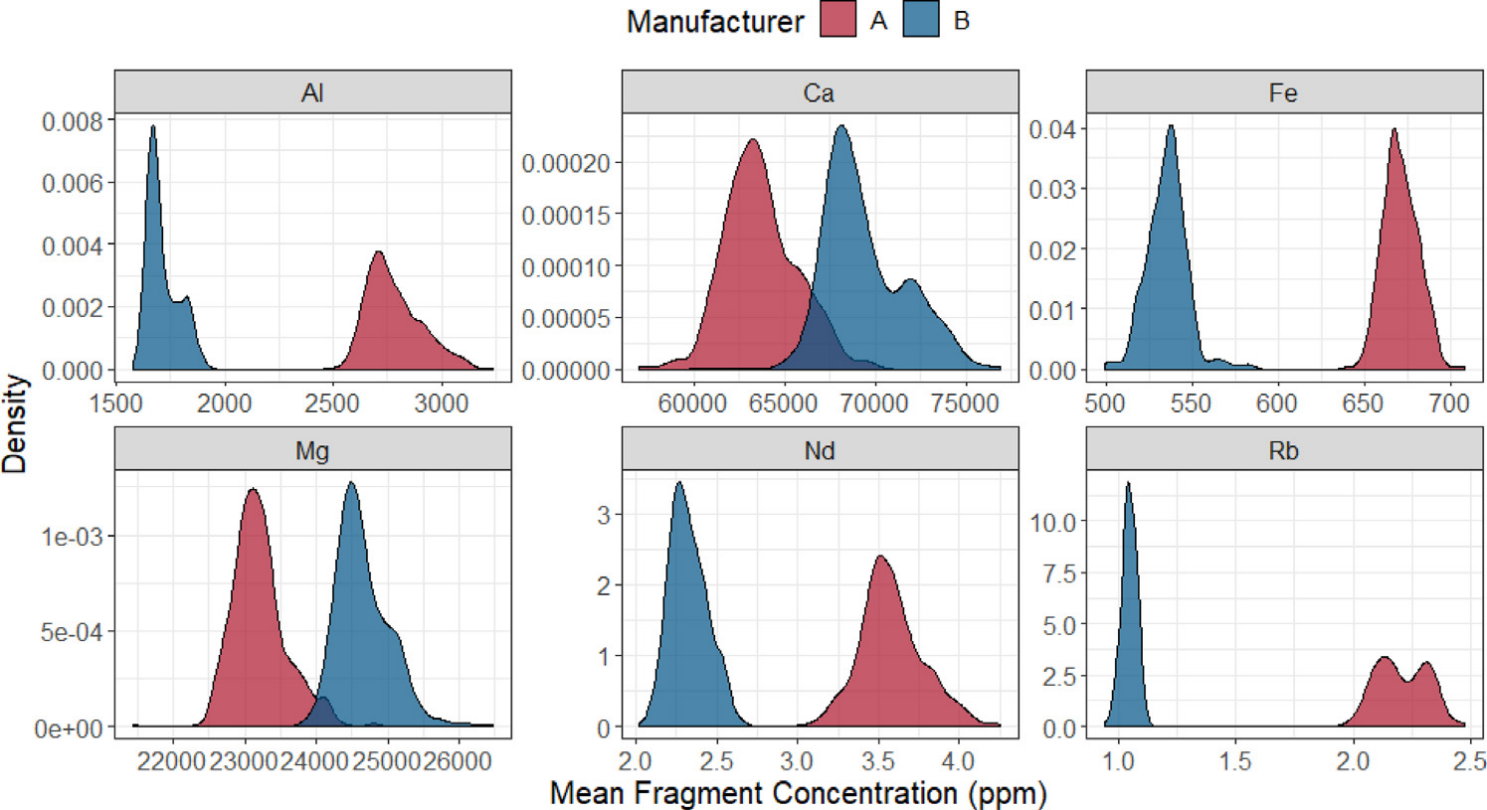
Density plots of measured fragment mean for selected element concentrations in the glass database.

**Fig. 2:**
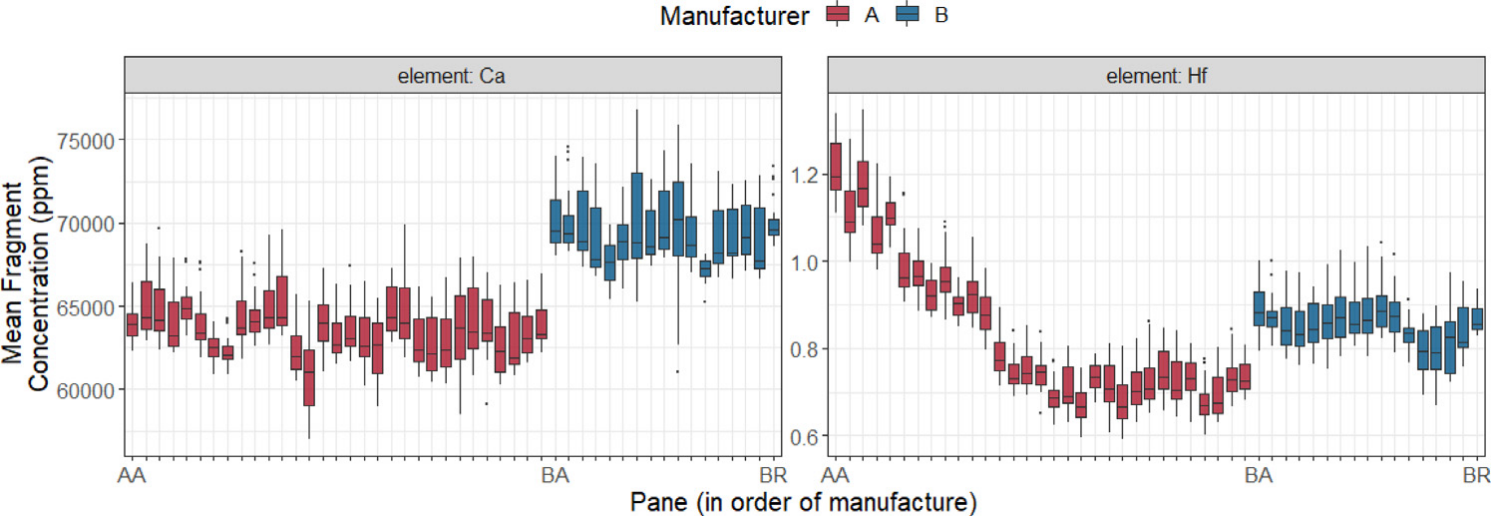
Box plots of mean fragment measurements within panes for trace elements Ca and Hf. The x-axis is ordered according to the manufacturer and date of pane manufacture.

The dataset (raw data) includes concentration measurements for 18 elements (in parts per million or µg g^−1^) in almost 8000 individual spot analyses from approximately 1200 fragments from 48 float glass panes produced by two glass manufacturers in the United States. In total, there are 48 Excel files with each file containing the concentration measurements from each of the 48 float glass panes. In each file from one pane, there are 165 rows of measurements and 22 variables containing company ID, pane ID, fragment number, replicate number, and concentration values of 18 elements. The 18 elements that were measured are: *Ca, Na, Mg, Al, K, Fe, Li, Ti, Mn, Rb, Sr, Zr, Ba, La, Ce, Nd, Hf, and Pb.* The unit for all measurements is parts per million (µg g^−1^). Table 1 shows the first four observations from fragment 1 from pane BA produced by company B. Fig. 1 shows empirical density plots of selected elemental concentrations (Al, Ca, Fe, Mg, Nd, and Rb) colored by manufacturers. These empirical densities summarize the range of values of elemental concentrations included in the database. Fig. 2 shows box plots of the mean of elemental concentrations of Ca and Hf within fragment within panes, colored by manufacturers. In the plots, panes are ordered by production dates.

**Table 1:** The first few observations in the glass data from pane BA produced by company B. The elemental concentrations are shown in ppm.

Company	Pane	Fragment	Rep	Li7	Na23	Mg25	Al27	…	Pb208
CompanyB	BA	1	1	2.13	100,240	24,770	1756	…	0.981
CompanyB	BA	1	2	2.46	100,630	24,640	1725	…	1.043
CompanyB	BA	1	3	1.94	100,310	24,880	1739	…	1.006
CompanyB	BA	1	4	2.16	101,780	24,970	1742	…	1.036

## 2 Experimental design, materials, and methods

### 2.1 Experimental design

Glass manufacturers A and B located in the Midwestern region in the United States donated the float glass samples for our study. Panes were about 25 squared inches in size and were harvested daily, from the left and the right sides of the ribbon.

The dataset (raw data) includes 31 panes of a float glass manufactured by Company A and 17 panes manufactured by Company B. Company A panes were labeled AA, AB, …, AAR and Company B panes were labeled BA, BB, …, BR. The panes from Company A were produced within three weeks (January 3–January 24, 2017) and the panes from Company B were produced within 2 weeks (December 5–December 16, 2016). The production date of each of the panes in the database is included in the data repository. In total, there are 48 Excel sheets containing 165 measurements of 24 fragments from each of the 48 panes.

Each sample pane was cut into half. The first half was kept in our lab for reference. The remainder half pane was broken to create random fragments. Among the broken fragments, we randomly select 24 fragments from each pane. Each fragment was measured five times repeatedly, for 21 of the 24 fragments in each pane; for the remaining three fragments in each pane, we obtained 20 replicate measurements. In some cases, a fragment may have fewer than five replicate measurements if the ablation spot was not optimal (e.g., hitting a fracture). Fig. 2 in Park and Tyner (2019) [4] shows an example scheme of how we get measurements from one pane.

### 2.2 Methods

Analytical procedures to carry out the measurements followed the protocols recommended by ENFSI [1] and ASTM ([2]). Each forensic glass sample was broken into four quadrants, and then six fragments from each quadrant were selected, making a total of 24 fragments per sample. Fragments from two samples were embedded in a 1″ diameter epoxy mount, avoiding external glass surfaces, and the mount was polished flat. Each mount was carbon-coated and then given a quick wipe, which removed the coating from the glass but not the epoxy. This provided a visual contrast between the glass and epoxy, which made it easier to navigate around the mount using the camera on the laser ablation unit to set points for analysis.

Trace elements in forensic glass fragments were measured by LA-ICP-MS at the University of Iowa using a 213 nm NewWave laser ablation unit. Analyses were made using He carrier gas and an 80 µm spot, ablating at 10 Hz for 50 s, following a 30 s baseline measurement, with a fluence of 14 J cm^−2^. A 45 s washout period was used between each analysis spot. Ablation products were analyzed with a Thermo X-series II quadrupole ICP-MS instrument. Tuning for maximum sensitivity and minimizing oxide and doubly-charged ion formation was done using the NIST612 glass, and the FGS-2 forensic glass standard was used to check that CeO/Ce was < 1%. The following isotopes were monitored during signal acquisition on the ICP-MS: lithium (^7^Li), sodium (^23^Na), magnesium (^25^Mg), aluminum (^27^Al), silicon (^29^Si), potassium (^39^K), calcium (^42^Ca and ^43^Ca), titanium (^49^Ti), manganese (^55^Mn), iron (^57^Fe), rubidium (^85^Rb), strontium (^88^Sr), zirconium (^90^Zr), tin (^118^Sn), barium (^137^Ba), lanthanum (^139^La), cerium (^140^Ce), neodymium (^146^Nd), hafnium (^178^Hf) and lead (^208^Pb). Data were processed with the Iolite software [5], using FGS-2 as the calibration standard (using values from [6]) and ^29^Si as the internal standard (assuming a value of 72 wt% SiO_2_ for all float glass samples and standards as recommended by Willis et al. [6] and ASTM [2]). The FGS-2 calibration standard was analyzed at regular intervals throughout each analytical session to correct for instrumental drift. NIST1831 and DGS-1 were analyzed as quality control standards during each analytical session. One glass pane sample, comprising 24 fragments, was analyzed per analytical session that lasted about 9 h. The unit for all concentration measurements is parts per million (ppm), which is equivalent to µg g^−1^. Note that for a few samples, the Pb concentration data are variable and elevated. This was due to an intermittent grounding fault with the ICP-MS instrument that only affected masses > 190.

Analytical sequence (standards and samples) used: a total of 203 individual spot analyses per sample.

Calibration std (FGS-2) × 2 spots
Fragment #1 × 5 spots
Fragment #2 × 20 spots
Calibration std (FGS-2) × 2 spots
Fragments #3 to #5, each × 5 spots
Data quality #1 (DGG-1) × 5 spots
Calibration std (FGS-2) × 2 spots
Fragments #6 to #9, each × 5 spots
Data quality #2 (NIST-1831) × 5 spots
Calibration std (FGS-2) × 2 spots
Fragments #10 to #13, each × 5 spots
Calibration std (FGS-2) × 2 spots
Fragment #14 × 20 spots
Data quality #1 (DGG-1) × 5 spots
Calibration std (FGS-2) × 2 spots
Fragments #15 to #18, each × 5 spots
Calibration std (FGS-2) × 2 spots
Fragments #19 to #22, each × 5 spots
Data quality #2 (NIST-1831) × 5 spots
Calibration std (FGS-2) × 2 spots
Fragment #23 × 5 spots
Fragment #24 × 20 spots
Calibration std (FGS-2) × 2 spots

## Appendix Supplementary materials

Image, application 1
